# Supplementary figures and images for: Structure-guided mutagenesis of the capsid protein indicates that a nanovirus requires assembled viral particles for systemic infection
Source: PLoS Pathog. 2023 Jan 9;19(1):e1011086. doi: 10.1371/journal.ppat.1011086 (PMC9858847; doi:10.1371/journal.ppat.1011086)

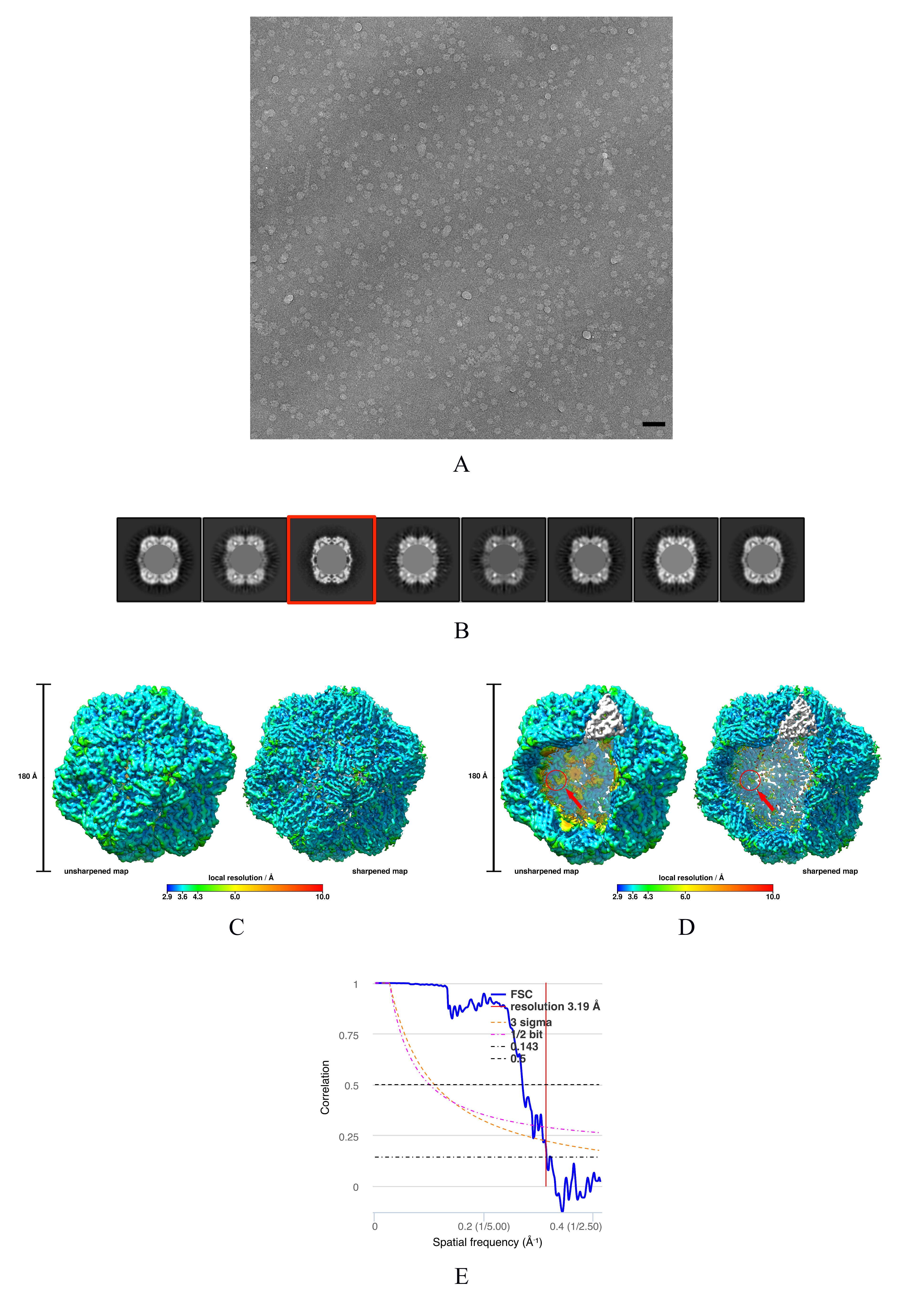

Supplement: S1 Fig — (A) A representative cryo-EM image of FBNSV viral particles (scale bar: 50 nm). (B) Central sections of FBNSV 3D classes. The central part of the particles was masked out. The particles contained in the 3D class marked with the red frame were used for the final 3D refinement. (C) FBNSV cryo-EM reconstructed density (EMD-10097). The two isosurfaces (unsharpened and sharpened map) are coloured according to the estimated local resolution. (D) Sectional view (one octant removed) of the unsharpened and sharpened densities. Colours as in C, except for a single CP subunit which is highlighted in white for reference. The position of a genome density residual is indicated by the arrows. (E) Fourier Shell Correlation curve (excerpt from the EMD-10097 validation report). (TIF) [file ppat.1011086.s001.tif]

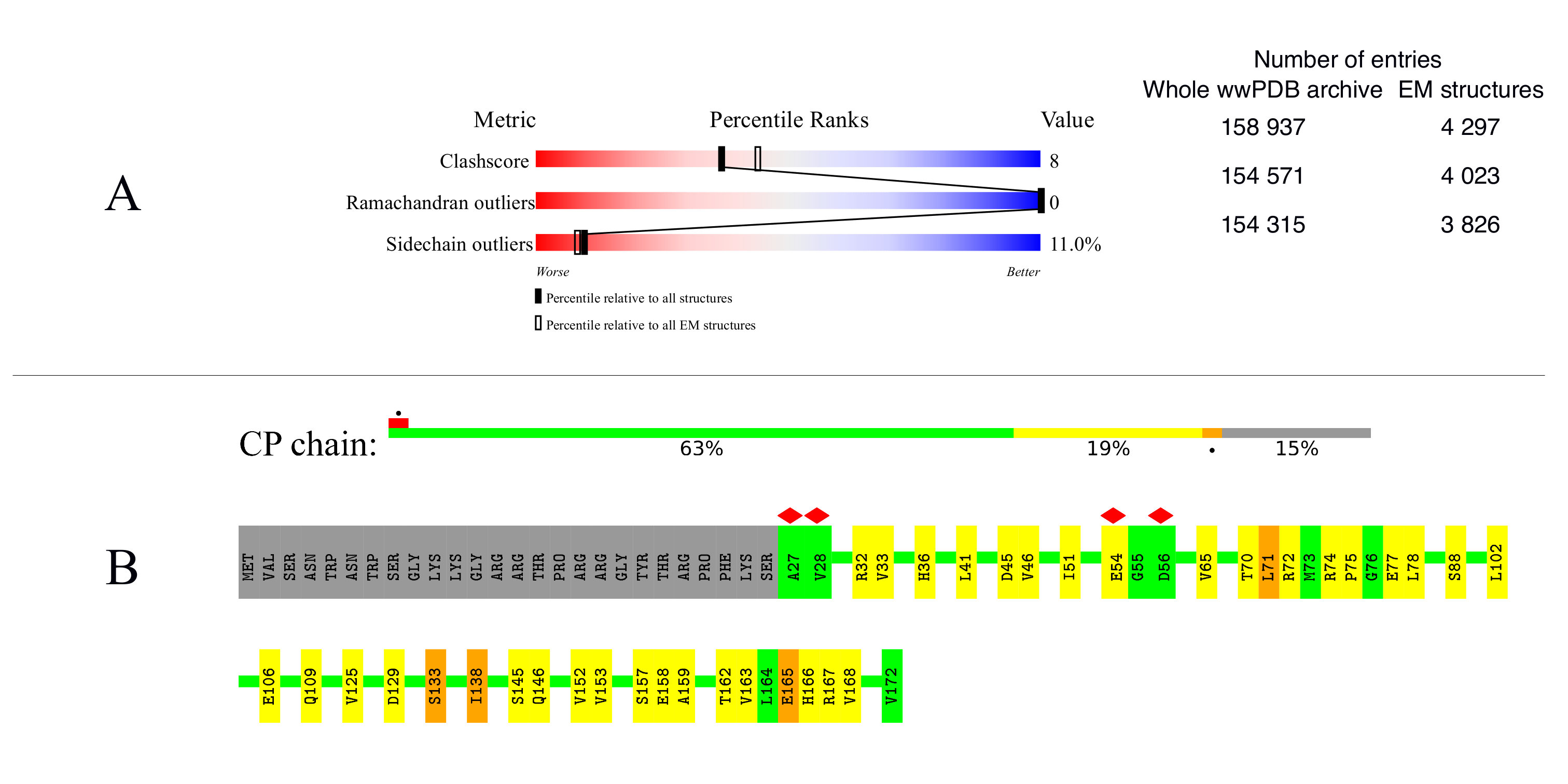

Supplement: S2 Fig — (A) Global validation metrics values, percentile scores (ranging between 0–100), and number of entries on which the scores are based. (B) Excerpt from the wwPDB validation report. The first (top) graphic for the CP chain summarises the proportions of the various outlier classes displayed in the second (bottom) graphic. A dot represents fractions ≤ 5%. The upper red bar indicates the fraction of residues that have poor fit to the EM map (all atom inclusion < 40%). The second graphic shows the sequence view annotated by issues in geometry and atom inclusion in map density. Residues are colour-coded according to the number of geometric quality criteria for which they contain at least one outlier: green = 0, yellow = 1, orange = 2 and red = 3 or more. A red diamond above a residue indicates a poor fit to the EM map for this residue (all atom inclusion < 40%). Stretches of 2 or more consecutive residues without any outlier are shown as a green connector. Residues present in the sample, but not in the model, are shown in grey. (TIF) [file ppat.1011086.s002.tif]

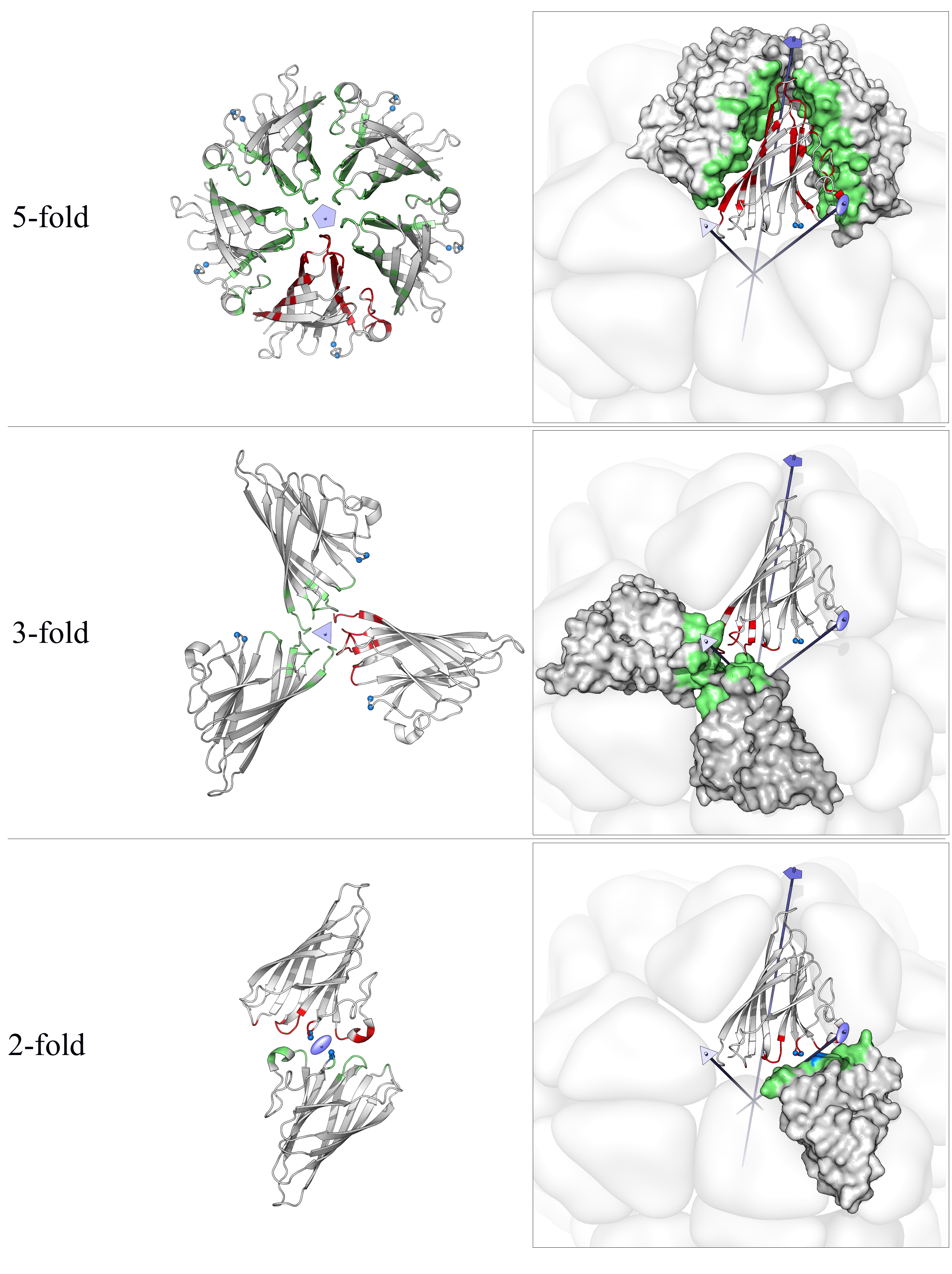

Supplement: S3 Fig — Zones involved in inter-subunit contacts are coloured green and red. The position of Ser 87 and Ser 88, which have been targeted for site directed mutagenesis, is indicated by the blue spheres. (TIF) [file ppat.1011086.s003.tif]

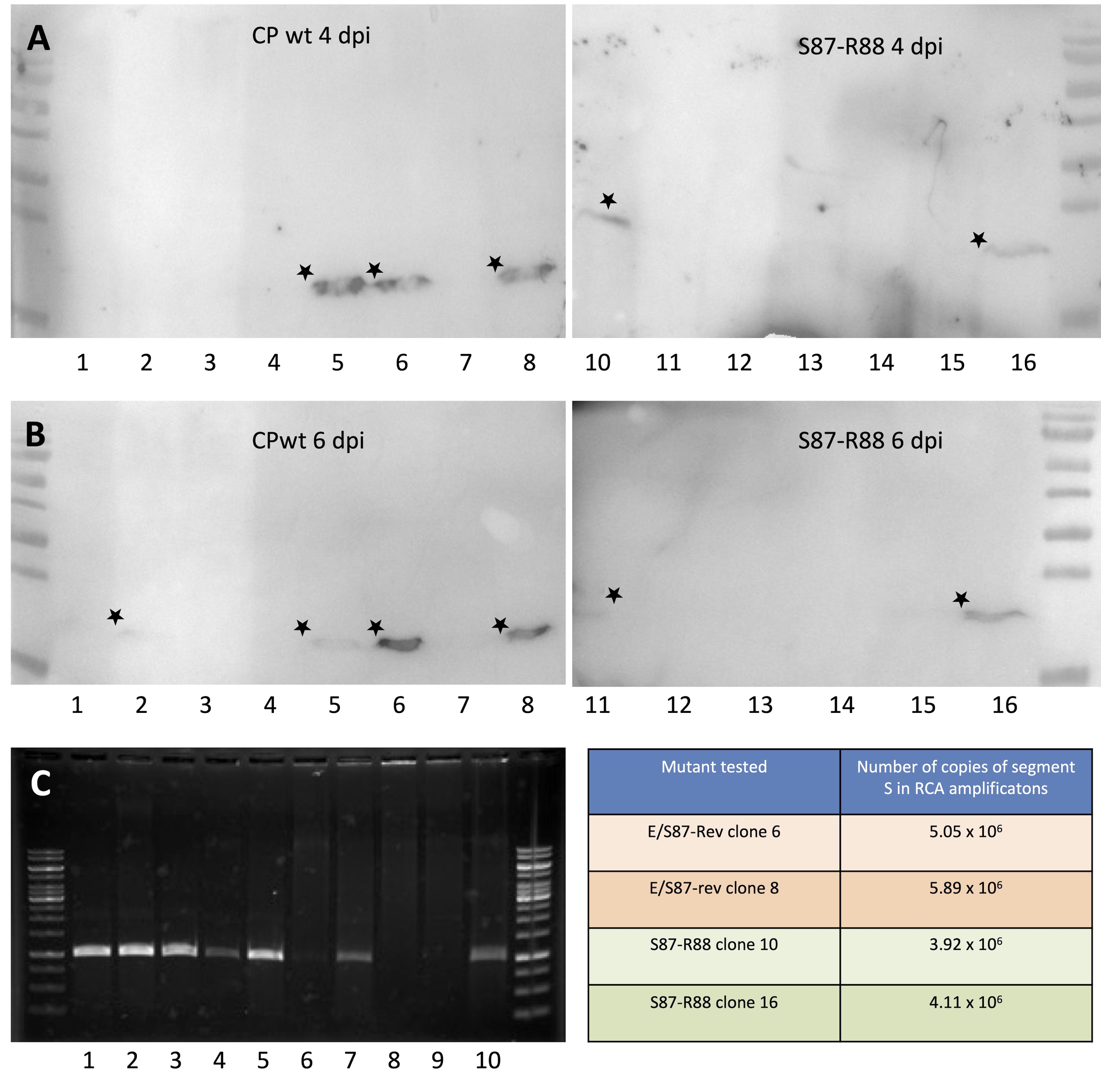

Supplement: S4 Fig — Two leaves per plant were agroinfiltrated as described in Materials and Methods. Two sets of eight plants were respectively infiltrated with wild type (N° 1 to 8) and mutated (N° 9 to 16) CP clones, together with the seven other segments. One of the two infiltrated leaves of each plant was collected 4 days post-agroinfiltration (A), and the other 6 days post infiltration (B). The CP protein detection is indicated with a small star in each lane. The various leaves expressed the CP protein inconsistently, and we have no explanation for this observation, other than a technical hurdle related to inefficient infiltration. It is also notable that the S87-R88 CP is expressed in a reasonable number of leaves but in amounts seemingly smaller than the wild type. We assume that failure of the mutated CP to assemble virus particle may affect its stability. The molecular weight markers are on the side of the gels and corresponds (from bottom to top) to 15, 25, 35, 55, 70, 100, 130 and 250 kDa. Plant N° 9 in A and B was negative and is not shown. Plant N° 10 died in between the two sampling dates. In an additional control experiment (C), we also verified that mutation of segment S did not impair its capacity to replicate. Total DNA was extracted from infiltrated leaves, submitted to RCA amplification and digested with AatII, as described in the Materials and Methods. In the agarose gel, the infiltrated segment S or derivative mutants are: line 1 = E/S87-Rev clone 6 (6 dpi), lane 2 = E/S87-Rev clone 8 (6 dpi), lane 3 = S87-R88 clone 10 (4 dpi), lane 4 = S87-R88 clone 11 (4 dpi), lane 5 = S87-R88 clone 16 (4 dpi), lane 6 = S87-R88 clone 10 (6 dpi), lane 7 = S87-R88 clone 15 (6 dpi), lane 8 = mock infiltrated leaf, lane 9 = leaf infiltrated with wild type FBNSV segments but omitting segment R, lane 10 = leaf infiltrated with all 8 wild type segments. Molecular weight marker on right and left is TrackIt 1 kb DNA ladder (Thermofisher Scientific). An RCA amplified band of approximately [file ppat.1011086.s004.jpg]
