# Supplementary material for: Structure-guided mutagenesis of the capsid protein indicates that a nanovirus requires assembled viral particles for systemic infection
Source: PLoS Pathog. 2023 Jan 9;19(1):e1011086. doi: 10.1371/journal.ppat.1011086 (PMC9858847; doi:10.1371/journal.ppat.1011086)
Supplement: Attachment — Submitted filename: FBNSV-PPATHOGENS.2-Responses-to-reviewers-SBSTPB.pdf [file ppat.1011086.s006.pdf]

Manuscript PPATHOGENS-D-21-01831.

Point-by-point response to reviewers' comments

We have numbered our responses in order to easily refer to some of them whenever several distinct reviewers comments are actually addressing the same point. The indicated lines refer to the revised version of the manuscript.

Part I - Summary
Please use this section to discuss strengths/weaknesses of study, novelty/significance, general execution and scholarship.

Reviewer #1: *This manuscript by Trapani et al. presents a report on the structure guided mutagenesis of Faba bean necrotic stunt virus (FBNSV) in an attempt to address the mechanism of intercellular transmission of the multi-partite genome. This works addresses an important question in the biology of Nanoviridae. Not being a structural biologist, I cannot critically judge the quality of the image reconstruction beyond the reported statistics which suggest the quality is good. A clear and fairly thorough description of the structural of FBNSV is provided, which revealed an interesting twist to the standard T1 arrangement of capsid proteins (CP). Using their structural data, the authors select positions for amino acid substitution with the intent of disrupting capsid assembly. Serine residues at positions 87 and 88 were selected and replaced by charged amino acids. Based on the structural data, this should destabilize the two-fold axes by creating a charge repulsion. To this point in the paper, the methods and conclusions are sound.*

Reviewer #2: *This is an interesting paper wherein a cryo-EM derived capsid structure of FBNSV is presented, compared with other similar viral capsid structures and used to manufacture several FBNSV coat protein mutants to test whether either virions or coat-protein nuclear protein complexes is the means by which FBNSV disease progression in plants is achieved. The mutations selected were predicted to allow for the formation of 5-fold axis of capsid symmetry thereby, hopefully, ensuring the formation of nuclear-protein complexes could occur but unfortunately no verification of this is presented. However, only one form of FBNSV mutant coat protein was detected by western blotting experimentation, in infiltrated leaves to reassure the reader but no coat protein binding data is presented. From this the authors conclude that their findings support the idea that disease progression is dependent upon assembled viral particles.*

Authors response N°1: We have carried out a key additional experiment to best support our conclusion. We have produced and purified recombinant versions of the FBNSV coat protein using a bacterial expression system, and observed the level of self-assembly by negative-stain electron microscopy. While the non-mutated version of the recombinant coat protein forms pentamers which further assemble into spherical virus-like particles (VLP), the version containing one of the designed mutations cannot assemble beyond the pentameric stage and no VLPs could be observed.
We believe this experiment confirms our conclusion that the engineered mutations of serines 87 and 88 hamper interaction at the two-fold axis and thereby prevent assembly of several pentamers into icosahedral particles. We thank the reviewers for prompting us to carry out this experiment. We initially thought it had little chances of success. However, by removing the charged N-ter stretch from the coat protein, we could finally observe VLP formation and this outcome now significantly enhances the soundness of our hypothesis. The related major modifications in the manuscript are in lines 333-355 (Results), 447-451 and 458-461 (Discussion), and 701-764 (Materials and Methods). An additional figure (Fig 6) is included (*Remark*: Fig. 6 from the previous manuscript has been renumbered to Fig. 7)

Reviewer #2: *A major issue with this study is the poor infiltration rate, hence infectivity, that is achieved to register virus infection and consequently an even poorer infection rate when the mutant coat protein forms are employed. This is brought home from the data in Table 3 and is commented upon in the legend to S4 Fig. Eight unique genomic segments are necessary for FBNSV infection and consequently have been cloned on separate binary plasmid vectors and therefore require eight independently transformed Agrobacteria for leaf infiltration to drive the subsequent viral infection. Surely to improve upon this, all eight unique genomic segments should be cloned into one binary vector and any mutated gene, for experimental analysis, simply replacing its wild-type version when necessary? Thus, infiltration experiments could be performed with one transformed Agrobacteria. It is noteworthy that complete sets of genomic segments rarely occur in infected plant cells, see abstract, but is this a consequence of the infiltration procedure?*

Authors response N°2: Yes, unfortunately, agroinfiltration of eight independently-transformed agrobacteria does result in an infection rate that is highly variable and may be low, ranging from 10 to 60 % depending on time of the year and other unknown factors. This is a heavy burden we have to cope with, and so do the other laboratories that have worked or still work with this type of nanoviral infectious clones, the only type of infectious clones available thus far. The low success of systemic infection of plants with the eight genomic segments implies that we manipulate and agroinoculate large numbers of host plants to get sufficient infected ones, which is what we systematically do. The bottom line of our experiment reported in Table 3 is that some host plants are indeed repeatedly infected when wild type coat protein segment is present in the inoculation trials, but never when any of the serine 87 and/or 88 are mutated. We have compensated the inevitable low infection rate of this agroinfiltration technique by a large number of plants and by 2 to 4 experimental repeats for each of seven mutants. With that effort, we think this comment from reviewer 2 is unfair

The "all-segments-in-one-clone strategy" has been attempted by several laboratories working on *Nanoviridae*, including ours. The release of a segment from a bacterial plasmid and its replication in planta requires that it be flanked by the segment stem-loop origin of replication on both sides (see ref Grigoras *et al.* 2009), which implies a redundant stem-loop sequence even if only one segment is cloned. Cumulating several segments within the same clone means introducing several times this conserved stem loop origin of replication, and this gives rise to unstable plasmids which immediately delete most of the viral sequences. What is proposed by Reviewer 2 as a simple solution is actually impossible, or it does seem so after repeated attempts carried out by at least four laboratories in France, Germany, and Australia during many years.

The published report that distinct viral segments accumulate in distinct plant cells (Sicard *et al.* eLIFE 2019) has nothing to do with the inoculation of eight agrobacteria clones. This "pluricellular" phenomenon is systematically observed in infected plants, whether inoculated by agroinfiltration of infectious clones or more naturally form plant-to-plant by aphid vectors.

Reviewer #2: *Nevertheless, the structural investigation is sound and with text modifications this manuscript should, in due course, be acceptable for publication.*
Points to note –
I had difficulty following the text that has become incorporated with its figure legends. This hindered my understanding of the investigation presented in the manuscript.

Authors response N°3: We have moved the figure legends to the end of the manuscript

Reviewer #2: *The 25 residue "EF connection" is mentioned prominently (page 12) but there is no clear explanation as to what exactly this is and its relevance to the study?*

Authors response N°4: This region comprises residues 87-111 (i.e. from the first residue following beta-strand E to the last residue preceding beta strand F, see Fig. 3) and can be identified in Fig 2D by spotting the end of strand E and the beginning of strand F at the bottom of the jelly roll:

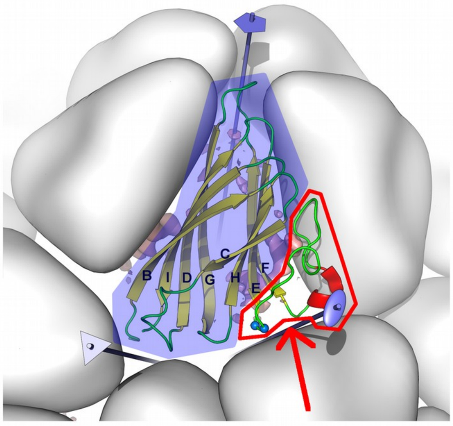

D

Centre de Biologie Structurale – Inserm U1054, CNRS UMR 5048 , Univ Montpellier
29 rue de Navacelles - 34090 Montpellier
Tel: +33 (0)4 67 41 77 29 – Fax: +33 (0)4 67 41 79 13 – E-mail: stefano.trapani@cbs.cnrs.fr

As mentioned in the text, this region: i) contains the two mutated residues (Ser 87 and Ser 88) important for formation of full icosahedral assemblies (lines 227-228); ii) participates, downstream, to the fivefold contacts that stabilise the pentameric capsomeres (lines 219-223); iii) contains residues 102 to 104 which are in close contact with the observed residual DNA density (lines 250-253). Also (not stated in the text) this stretch is much longer as compared to viruses like BFDV, SPMV and STMV which organise their icosahedral inter-subunit contacts in a prominently different manner (see Fig 7).

<u>Reviewer #2:</u> *The lack of data concerning the binding of a genomic segment to wild-type and mutant coat protein is an omission that should be corrected (page 24).*

Authors response N°5: We have not directly tested the interaction between viral ssDNA segments and coat protein mutants and wild type because the mutated residues are not in contact with the observed residual genomic density at the fivefold interfaces. Moreover, the additional experiment with bacterially expressed mutants suggest that the fivefold interface is not affected, since they still form pentamers. This is now explicitly stated in Results (lines 349-352) and Discussion (lines 447-454).

<u>Reviewer #2:</u> *How do we know that the prediction concerning virus particle assembly, stated on page 15 (line 321) to be true?*

Authors response N°6: Since we think this is the major criticism to our original manuscript, we have answered this important point in our response N°1

<u>Reviewer #2:</u> *Table 3. Ideally a positive control for infectivity should be 100%. At best it was 22/48 or 45%. At worse it was only 7/96 or 7% for all eight genomic segments of the original pBin19 infectious clones. It would be ideal if these rates could be improved upon if only to strengthen the conclusions drawn from the study.*

Authors response N°7: This point is answered in detail in our response N°2 to a very similar comment from the same reviewer. We do not understand why the positive control should be 100 %. This is nearly never the case when inoculating a plant virus, and the infection rate —depending on the technique of artificial inoculation or the natural transmission route— is very frequently low. If really required by Reviewer #2, we can cite tens of papers where the inoculation of an infectious viral clone with a wild type sequence or even natural transmission by vector is very far from 100 %. In our point of view, the crucial point here, as already mentioned above, is that we carried out two to four experimental repeats for each of the 7 mutants produced and, in all trials, we always got plants infected by the wild type sequence and never by the mutated sequences.

<u>Reviewer #3:</u> *The manuscript 'Structure-guided mutagenesis of the capsid protein indicates that a nanovirus requires assembled viral particles for systemic infection' by Trapani et al. present new and interesting results regarding the multipartite ssDNA virus Fava bean necrotic stunt virus (FBNSV), a species in the genus Nanovirus(family Nanoviridae). This virus has a genome composed of 8 circular ssDNA segments that are individually encapsidated into spherical virions that measure ~18 nm in diameter. The two main results presented in thre present study are 1) a detailed analysis of the virion structure via cryo-EM and atomic modeling of purified particles and 2) the prediction and experimental confirmation that amino acid residues Ser 87 and Ser 88 play a key role in virion formation and systemic infection. The work is well-done and the results support most of the conclusions. However, some additional experimental evidence could strengthen some conclusions. Also the manuscript could be shortened substantially and the writing improved in some places. Thus, this work warrants publication, but I am not certain it is sufficient to warrant publication in PLoS Pathogens.*

Authors response N°8: We deeply hope that with the additional experiments and the modifications of the manuscript in response to all reviewers' comments, our work will now be judged acceptable for publication in PLoS Pathogens

Centre de Biologie Structurale – Inserm U1054, CNRS UMR 5048 , Univ Montpellier
29 rue de Navacelles - 34090 Montpellier
Tel: +33 (0)4 67 41 77 29 – Fax: +33 (0)4 67 41 79 13 – E-mail: stefano.trapani@cbs.cnrs.fr

Reviewer #4: *The paper reports the first atomic structure and structure-based replication of Faba bean necrotic stunt virus (FBNSV), a model virus of the Nanoviridae family. Like other members of the Nanoviridae, FBNSV is a multipartite ssDNA genome (+) of about 8Kb, composed of 8 circular segments. Each genomic segment is about 1kb in size and is packaged separately in a T=1 capsid, necessitating a total of 8 capsids for the package and transmission of the entire genome. How complete sets of genomic segments complement each other in host cells for productive infection remains controversial. In this work, the authors first determined a near-atomic resolution (3.2 Å) structure of the capsid from about 5000 cryo-EM capsid particles from, revealing well-conserved jelly-roll fold in its capsid protein (CP), CP-CP interactions and protein-DNA interactions. Based on the cryo-EM derived atomic model of the capsid, several residues involved in capsid protein (CP) inter-subunit interactions and CP-DNA interactions have been identified. A series of site-directed mutations at the CP subunit-subunit interfaces at the 2-fold icosahedral axis have been designed in order to prevent the formation of fully assembled particles. These mutations systematically suppress FBNSV infectivity. These observations were interpreted as to support of the two existing mechanisms of nanovirus replication across host cells—the viral genome does not propagate within the plant vascular system under the form of uncoated DNA molecules or DNA-CP complexes, but rather outspreads as assembled viral particles which appear indistinguishable with respect to their DNA content. Overall, the paper touches upon an important and controversial issue regarding DNA genome packaging of multipartite ssDNA viruses.*
However, the small dataset included in the cryo-EM analysis has unfortunately prevented definitive conclusion regarding CP-DNA interactions.

Authors response N°9: See author response N°17

Reviewer #4: *It would be also desirable to determine the impact of the CP mutations on capsid assembly.*

Authors response N°10: This important recurrent comment from the reviewers is answered in our response N°1.

Part II – Major Issues: Key Experiments Required for Acceptance
Please use this section to detail the key new experiments or modifications of existing experiments that should be absolutely required to validate study conclusions.
Generally, there should be no more than 3 such required experiments or major modifications for a "Major Revision" recommendation. If more than 3 experiments are necessary to validate the study conclusions, then you are encouraged to recommend "Reject".

Reviewer #1: *Infectivity data for WT and variant form of FBNSV show that the amino acid substitutions impair propagation of the virus. I agree with the authors that the absence of intact capsids does correlate with the lack of propagation. However, simply breaking a biological system cannot be equated with causation. For example, how can the authors rule out changes in the behavior of the mutated genome segment rather than capsid assembly? A straightforward control would to generate silent mutations that alter the genome sequence without changing the sequence of the CP. Perhaps this data is already available in the form of spontaneous reversions that rescued infectivity.*

Authors response N°11: We believe that our parsimonious interpretation of the results is very classical and represents the most probable scenario: serine 87 and 88 mutations hinder CP assembly beyond the formation of pentamers (now directly demonstrated by bacterially expressed CP and mutants) and abolish systemic infections of host plants, suggesting that full assembly is required for infection. Of course one can always propose alternative interpretations, but we did not find any that appeared sound enough to be mentioned. The very multipartite nature of FBNSV implies that very different sequences of the 8 segments are all efficiently replicated, encapsidated, moved long distance and aphid-transmitted. Keeping this in mind, we can hardly imagine that point mutations in the CP could make the sequence (the segment) itself behave totally differently.

Centre de Biologie Structurale – Inserm U1054, CNRS UMR 5048 , Univ Montpellier
29 rue de Navacelles - 34090 Montpellier
Tel: +33 (0)4 67 41 77 29 – Fax: +33 (0)4 67 41 79 13 – E-mail: stefano.trapani@cbs.cnrs.fr

We do not wish to go along the alternative interpretation from Reviewer #1 because it is not specific and thus we do not see how we could test for it, or what we should test for. First, if we do not propose a hypothetical default for the mutated segment (other than assembly default) then we cannot propose an experiment directly testing for this default. Second, as suggested by Reviewer #1, if we create a silent mutation at the same serine 87 and/or 88 codon position, we will generate a mutant that will be different from the ones we tested, and there will be no definitive evidence that the behaviour of this novel mutant actually recapitulate the behaviour of our non-synonymous serine mutants. So we have the feeling that this experiment would not respond to the comment; i.e. this new silent mutant may have no effect while our serine mutant may still have the "cryptic" effect evoked by Reviewer #1.
In conclusion, we feel that the way we interpret our result is very classical, logical and most parsimonious and we would like to maintain the corresponding text as it is.

Reviewer #1: *A second critical point is that both arguments put forth in favor of the random propagation model are not well formulated. Lines 528-529 - Did the authors confirm that all genome segments were present in purified virus samples used in the image reconstruction? Without that data, such a conclusion is not warranted.*

Authors response N°12: We have tried to improve the formulation of the random propagation in the new version of the text (lines 479-490 and 502-510).
We apologise for omitting the information on the frequency of the distinct segments in the viral population analysed in the previous version of our manuscript. Since the yield of FBNSV purification is so low (a few micrograms of virus particles per kilo of infected plant material), numerous infected plants are pooled and extracted. We obtain then a suspension of virus particles where the frequency of each segment is an average of the frequencies in individual infected plants. Typically (see Sicard *et al.* 2013, cited in the manuscript) some segments are rare (e.g. C, M, S and R) and others are more frequent (e.g. N, U1, U2 and U4). We have used an aliquot of the virus particle suspension analysed by Cyro-EM and estimated the relative frequency of each segment by qPCR. The frequency of segments C, M, N, R, S, U1, U2 and U4 is respectively 4.25, 7.83, 15.58, 3.33, 6.84, 11.56, 20 and 30.61 %. This additional data is now included in the new version of the manuscript (lines 136-143 and 625-627).

Reviewer #1: *The second point put forth is that structural differences were not seen in the EM data. This could be the result of: not all genome segments being significantly represented in the population (see above).*

Authors response N°13: See the response N°12 above

Reviewer #1: *Also, it is unclear to this reviewer why not being able to detect structural differences favors the random propagation model. This argument should be clarified.*

Authors response N°14: Our argument is actually trivial. There are two ways this virus could specifically sort segments and form complexes containing at least one copy of each. The first one would be by specific DNA-DNA inter segment interactions, but this is not likely if the viral genetic material is moving long distance in the host plant encapsidated into fully assembled virus particles. The second possibility would then be the existence of structural differences for particles containing different segments. Specific sorting/grouping of particles could thus be achieved according to these structural differences, which could thus represent a structural signature of the identity of the contained segment.
We hope text of the new version of the manuscript is clearer on this point (lines 479-490 and 502-510).

Reviewer #1: *Finally, the absence of evidence is not evidence of absence. There are comparisons of structural models between infectious virions and virus-like particles (packaging heterologous nucleic acid) that also fail to show differences in the overall capsid structure or at the interior CP surface in contact with nucleic acids, however, the capsids show different properties in solution (example reference: Tihova M et al. J Virol. 2004 Mar;78(6):2897-905. doi: 10.1128/jvi.78.6.2897-2905.2004. PMID: 14990708; PMCID: PMC353755)*

Centre de Biologie Structurale – Inserm U1054, CNRS UMR 5048 , Univ Montpellier
29 rue de Navacelles - 34090 Montpellier
Tel: +33 (0)4 67 41 77 29 – Fax: +33 (0)4 67 41 79 13 – E-mail: stefano.trapani@cbs.cnrs.fr

Authors response N°15: We agree with Reviewer #1 that similar outer surface structure does not necessarily mean similar physico-chemical properties and behaviour in solution. In fact the absence of obvious structural difference depending on the encapsidated segment is just one additional argument (not in itself fully conclusive) adding up to other evidence from other approaches supporting the random propagation model. This is now more cautiously discussed, in the context of these other converging evidences (lines 473-510)

Reviewer #3: *In particular, I would have liked to see the authors at least assess infiltrated leaves as an assay for virion formation. This would also involve monitoring viral replication and capsid protein (CP) expression with qPCR and Western blot analysis (Suppl Fig. 4), and then harvesting sufficient infiltrated leaf tissue for virion purification as described. This assay could be used to provide experimental evidence that a mutant is deficient or altered in virion formation. Aphid transmission is mentioned (lines 111-112), and this also could have been used as another line of evidence for lack of virion formation.*

Authors response N°16: Leaf-infiltration assays are very inefficient in terms of replication and protein production, perhaps because they are in mesophyll cells rather than phloem companion cells, which are the natural site of FBNSV infection. While leaf-infiltration assay can yield enough protein for detection by western blot, it is not imaginable to purify viral particles from such a little amount of infiltrated tissues and such a low viral accumulation therein (please also see Response N°12). Therefore, we have carried out additional experiments following a different direction. We have shown that mutated coat protein cannot assemble virus particles through expression in bacterial system (Response N°1)

Reviewer #4: *1. CP-DNA interactions. The data set used for the final reconstruction, ~5000, is rather small according today's cryo-EM standard, leading to inadequate analysis of 3D classification and asymmetric reconstructions for reliable conclusion regarding the status of sDNA genomic segments (lines 305-313). As a result, the CP-DNA interactions (Fig. 5A-B) can't be interpreted. I am not convinced by the modeled nucleic acid in Figure 5C-F .*
Based on the amount of virus samples obtained and the excellent concentration obtained in the cryo-EM images (fig S1A), it should have been relatively straightforward to obtain the required amount image data to perform such analyses for conclusive determination of CP-DNA interactions.

Authors reponse N°17: We partially agree with reviewer. The final number of particles included in the reconstructed map is rather classic for icosahedral viruses. Indeed, if one takes into account the symmetry-related views, a total number of 300,000 (=5000×60) different views are included in the final map, which explains why we reach a quite interesting resolution. The intensity of additional densities is strong, at least as strong as densities of the capsid shell, suggesting these CP-DNA contacts are present in most of the viral particles, whatever the DNA segment present. Figure 5C-F is an interpretation of these DNA-CP contacts. The high number of charged capsid residues involved in these contacts is consistent with the presence of nucleic acids. Since each DNA segment has a different nucleotide sequence, and since the reconstruction procedure used averages a many different (with respect to their DNA content) viral particles, it is not surprising that this region is not perfectly resolved.
We also agree that a higher number of viral particle would allow us to perform the analysis without imposing symmetry, and it could be interesting to better resolve these CP-DNA contacts. However, in our experience in EM and image processing, this is a huge job with a lot of uncertainty, because it would suppose to differentiate between DNA sequences.

Reviewer #4: *2. My main concern is that the impact of the mutations to capsid formation was assumed to be disruptive, but has not been experimentally established. The authors have demonstrated in S4 figure that CP proteins were expressed in both mutant and wild-type S inoculation experiments though at unexplained variable levels across different experiments. Efforts should be made to see what type of particles do these CP form—CP monomers, pentamers or capsids? Since the mutations only impact 2-fold interfaces, pentamers are expected based on Table 2.*

Authors response N°18: Again, this question is really important and was raised by all reviewers. As already indicated in our Response N°1, we have shown —using purified, recombinant versions of the capsid protein observed by negative-stain electron microscopy — that the non-mutated coat protein assembles into

Centre de Biologie Structurale – Inserm U1054, CNRS UMR 5048 , Univ Montpellier
29 rue de Navacelles - 34090 Montpellier
Tel: +33 (0)4 67 41 77 29 – Fax: +33 (0)4 67 41 79 13 – E-mail: stefano.trapani@cbs.cnrs.fr

spherical virus-like particles (VLP), whereas the recombinant protein containing one of the designed mutations does indeed form pentameric structures but not full VLPs.

Part III – Minor Issues: Editorial and Data Presentation Modifications
Please use this section for editorial suggestions as well as relatively minor modifications of existing data that would enhance clarity.

Reviewer #1: *Line 141 awkward sentence*
Authors: sentence modified

Reviewer #1: *Line 145 "can be" should be replaced with "were".*
Authors: done

Reviewer #1: *Tense issue Fig 2 panel C.*
Authors: We are sorry we cannot see the mistake.

Reviewer #1: *The ribbon diagram is difficult to see.*
Authors: This may be due to loss of image quality during conversion of the submitted images to the merged pdf. High-resolution images have been submitted and we hope they are and will be available to reviewers for download (it seems that clicking on the links in the PDF document allows downloads). We do not think that the problem comes from the original image.

Reviewer #1: *Fig 3 resolution is poor. (Other figures as well)*
Authors: This problem is likely similar to the previous one. We hope the downloadable high-resolution images will look better.

Reviewer #1: *Table 1. extra forward slash in Electron exposure row.*
Authors: There is no extra forward slash. Units are (electrons/Å^2) and the numerical value reported on the second column corresponds to (Electron exposure)/(electrons/Å^2).
We have modified the table by moving the units to the second column. We hope that the table is more easily readable now.

Reviewer #1: *Table 2 Probability needs to be explained*
Authors: This is described in details in: Krissinel E. Journal of Computational Chemistry. 2010;31: 133–143. doi:10.1002/jcc.21303
We have added this reference to the reference list.

Reviewer #3: *Line 25. Here it would be good to simply state the virions are spherical or icosahedrons measure 20 nm in diameter.*
Authors: The sentence has been modified. A diameter of ≈18 nm is reported.

Reviewer #3: *Line 29. Here and elsewhere. Based on knowledge of macromolecular trafficking in plants, it seems unlikely that viral proteins or mRNAs (very large molecules) could simply diffuse between cells. It might be better to state that it occurs by a yet to be identified means of cell-to-cell trafficking. It should also be noted that FBNSV is phloem- limited, so this means the cells involved would be those of the phloem.*
Authors: The text has been accordingly modified (lines 31-32): "...translocation of the gene products, through unknown molecular process".

Reviewer #3: *Lines 32-34. Actually, not much information is provided about 'how FBNSV genome segments propogate within the vasculature of the host plants for systemic infection', whereas the mutational analysis provided evidence that virions are the form in which the virus moves long distance in the phloem.*
Authors: We have modified the text as follows "Here, we question the form under which FBNSV genetic material propagates long distance within the vasculature of host plants…." (lines 34-36)

Reviewer #3: *Line 37. Suggest 'predicted' rather than 'designed'*

Centre de Biologie Structurale – Inserm U1054, CNRS UMR 5048 , Univ Montpellier
29 rue de Navacelles - 34090 Montpellier
Tel: +33 (0)4 67 41 77 29 – Fax: +33 (0)4 67 41 79 13 – E-mail: stefano.trapani@cbs.cnrs.fr

Authors: we think that, in view of our observations on the recombinant CP constructs, we can leave "site-directed mutations *designed* to prevent capsid formation".

Reviewer #3: *Lines 37-38. This sentence could be improved and made more accurate, as the term 'suppress FBNSV infectivity' is not clear. In fact, as noted for the agroinfiltrated leaves, it is likely that the virus replicated, particularly given that CP expression was detected by Western blot analyses (Suppl. Fig. 4). Thus, it seems more accurate to say that long distance movement was abolished.*

Authors: Yes we do agree with Reviewer #3 on this point and the text has been accordingly changed (lines 40-41).

Reviewer #3: *Lines 41-42. Make it 'in the form of virions (virus particles)'*
Authors: sentence modified (lines 43-46)

Reviewer #3: *Lines 43-46. This statement, while probably true, seems more appropriate for the discussion.*
Authors: we suppressed this statement from the Abstract.

Reviewer #3: *Lines 103-104. See comments above that it is highly unlikely that these gene products or mRNAs are diffusing among cells. In fact, it could be argued that all the virions and seqments concentrate in the shoot and root apices infecting progenitor phloem cells and allowing greater chance of multiple seqments infecting single cells.*
Authors: While we have changed "diffusion" to "translocation", the rest of this sentence (lines 97-100) is unchanged because it is simply reporting the conclusion from a previously published paper (Sicard *et al.* 2019). While the point of view of Reviewer #3 on the conclusion of this previous paper is interesting —and we would love to further argue on this— it is not the scope of the present manuscript.

Reviewer #3: *Line 117. Does several mean that there were mutants in addition to Ser 87 and Ser 88 used in this study?*
Authors: No. In order to avoid confusion, "several residues" has been replaced by "the residues" (line 112) and "mutations at **the** CP subunit-subunit interfaces" has been replaced by "mutations at **one of the** CP subunit-subunit interfaces" (line 114).

Reviewer #3: *Lines 124-125. As noted above, the phrase 'systematically suppress FBNSV infectivity' could be stated more accurately.*
Authors: the text has been accordingly modified to mention that what is abolished is long distance movement

Reviewer #3: *Line 127. Replace 'outspreading' with 'moves long distance'*
Authors: done

Reviewer #3: *Line 137. Indicate =~18 nm*
Authors: done

Reviewer #3: *Lines 140-141. What other region would be looked at?'*
Authors: The inner regions where the DNA segments are. The sentence has been slightly modified to make it clearer (lines 149-152).

Reviewer #3: **Line 150. Is penton the best term? It is the name of a company and a protein produced by adenoviruses.**
Authors: We replaced "penton" by "pentameric capsomere" everywhere in the text.

Reviewer #3: *153-154. Could immunosorbent EM be used to resolve this question?*
Authors: We agree with the reviewer. It could be a possibility to resolve this question. However, due to the time required to perform this analysis, which does not answer a crucial question regarding the aim of our study, we will not engage effort in that way. We observed that viral particles can loose pentameric capsomeres. This is the important piece of information here.

Reviewer #3: *157. Should 'loosing' be 'losing'?*
Authors: yes

Centre de Biologie Structurale – Inserm U1054, CNRS UMR 5048 , Univ Montpellier
29 rue de Navacelles - 34090 Montpellier
Tel: +33 (0)4 67 41 77 29 – Fax: +33 (0)4 67 41 79 13 – E-mail: stefano.trapani@cbs.cnrs.fr

Reviewer #3: *Lines 157-158. How well resolved was the loss of pentons?*
Authors: The observation of loss of pentons is derived from the observation that 2D class averages clearly outline globular domains that we assigned to pentameric capsomers once we computed the final 3D EM density map. In 2D classes, we clearly see these globular domains and a loss of some of them in some 2D classes. This is a qualitative evaluation of the loss of pentons. The resolution of pentons is related to the binning of images here, binning that was 2 (2.42 Å/pix). So we cannot expect to see finer details than 5 Å.

Reviewer #3: *Fig. 2B. It is hard to understand the context of Fig. 2B*
Authors: Some residues are labelled for reference. The atomic model and the density map are available for download.

Reviewer #3: *Line 209. Indicate the positively charged amino acid in the N terminus amino acid sequence Fig. 3.*
Authors: done.

Reviewer #3: *Line 210 and elsewhere. It would be good to better define ordered and disordered*
Authors: Disordered regions: dynamically flexible regions which are not static in solution. The prediction method used to detect such regions is described in : doi:10.1093/bioinformatics/bth195. A reference to that publication has been added in the revised version of the manuscript. "Disordered" has been replaced by "dynamically flexible" int the text.

Reviewer #3: *Table 1. Would this be better as a supplemental Table?*
Authors: We do not think so.

Reviewer #3: *Line 271. What is meant by 'biological assembly'?*
Authors: For clarity, we have replaced (in Table 2 legend) the expression "biological assembly" with "assembly of folded macromolecular chains in aqueous solution".

Reviewer #3: *Lines 327-328. How was this determined? Were residues specifically involved in DNA binding identified?*
Authors: We have changed the sentence "the mutated residues are not *predicted to interact with genomic DNA* at the fivefold interfaces" to "the mutated residues are not *in contact with the observed residual genomic density* at the fivefold interfaces" (lines 282-283). We think that the modified sentence helps to respond to a major comment above.

Reviewer #3: *Line 329-331. Suggest saying 'Single and double mutants were generated in the S-segment and fava bean plants agroinoculated with these mutants and the other seven segments.'*
Authors: The sentence has been modified (lines 284-286).

Reviewer #3: *Lines 332-334. This sentence could be improved. First, technically the mutant segment did systemically infect a plant (R87-S88). Second were a 'high number' of mutants and repeats really used? In the case of repeats, there were only 2 repeated experiments for each mutant(s), so the number was actual low (though a large number of plants were inoculated)*
Authors: This sentence has been changed to "None of them could ever systemically infect faba beans, despite the high number of inoculated plants per experiment and the number of experimental repeats (two to four) per mutant." (lines 287-289)

Reviewer #3: *Line 338. Suggest adding ..'which presumably occurred at the site of agroinoculation.*
Authors: This proposition has been added (lines 294-295)

Reviewer #3: *Table 3. This Table can be condensed by combining the repeated experiments*
Authors: We believe it is important to realise that the inoculation of control wild type coat protein segment together with the seven other genome segments always yielded infected plants, in every single experimental trial (even though the infection rate can be low in some of them) while the inoculation of mutated coat protein segment never did. Condensing the Table would hide this piece of information and thus we prefer to

Centre de Biologie Structurale – Inserm U1054, CNRS UMR 5048 , Univ Montpellier
29 rue de Navacelles - 34090 Montpellier
Tel: +33 (0)4 67 41 77 29 – Fax: +33 (0)4 67 41 79 13 – E-mail: stefano.trapani@cbs.cnrs.fr

maintain it as is. It is possible, however, would the Reviewers request it, to move this table to the Supplementary data.

Reviewer #3: *Line 352. Is 'no detectable wild type' correct? Isn't the revertants back to wild-type?*
Authors: Yes. The text has been replaced by "no detectable mutant sequence" (line 309). We apologise for this mistake.

Reviewer #3: *Line 356. Non-target better than fortuitous? Isn't it in the cloned S seqment rather than the plasmid?*
Authors: Yes, that is more accurate and the sentence has been amended accordingly.

Reviewer #3: *Line 371. Should this be infiltrated leaves?*
Authors: Yes, this is correct. we have modified this sentence accordingly.

Reviewer #3: *Line 394. Is 'incomplete' better than 'truncated'*
Authors: Yes. We have replaced 'truncated' with 'incomplete' everywhere in the text.

Reviewer #3: *Line 399. As compared with what?*
Authors: As compared to FBNSV. This is stated more clearly in the new version (lines 380-381).

Reviewer #3: *Line 484. Was the structure of the Maize streak virion also used in comparisons?*
Authors: No. That structure (doi:10.1006/viro.2000.0739) was solved at too low resolution (25 Å). Also, there is no atomic model available for that structure in the PDB.

Reviewer #3: *Line 489. Delete 'totally'*
Authors: done

Reviewer #4: *1. Lines 213-215: Accession codes should be listed at the end.*
Authors: According to PLOS Pathogens policy, accession codes are listed in "Data availability"

Centre de Biologie Structurale – Inserm U1054, CNRS UMR 5048 , Univ Montpellier
29 rue de Navacelles - 34090 Montpellier
Tel: +33 (0)4 67 41 77 29 – Fax: +33 (0)4 67 41 79 13 – E-mail: stefano.trapani@cbs.cnrs.fr
