# Supplementary material for: Structure-guided mutagenesis of the capsid protein indicates that a nanovirus requires assembled viral particles for systemic infection
Source: PLoS Pathog. 2023 Jan 9;19(1):e1011086. doi: 10.1371/journal.ppat.1011086 (PMC9858847; doi:10.1371/journal.ppat.1011086)
Supplement: Attachment — Submitted filename: PlosPath_FBNSV.pdf [file ppat.1011086.s007.pdf]

The study by Trapani et al., "Structure-guided mutagenesis of the capsid protein suggests that a nanovirus requires formed viral particles for systemic infection," provides novel and intriguing information on FBNSV. The paper reports the structure of the virion using cryo-EM, experimentally confirms the importance of residues Ser 87 and Ser 88 in the formation of dimers, and hypothesises about how systemic spread works. Despite how challenging it is to deal with ssDNA viruses from nanoviruses, the authors have done an admirable job of uncovering their structure and propagation. The work is well done, and most of the conclusions are supported by the findings. All of the significant issues were brought up by the prior reviewers, and the authors have convincingly responded. The only point I want to raise is that the structural work on BBTV CP (ref. 27), which reported similar structural results, needs mention where the authors compare FBNSV with AYVV (pages 19, 20). Yes, a lot of unanswered questions remain, and I am sure the authors will take them up subsequently. However, the present work is no less significant, and it definitely deems publication in Plos Pathogen. I congratulate the authors on their tremendous efforts in comprehending nanovirus capsid assembly.
