# Supplementary material for: Structure-guided mutagenesis of the capsid protein indicates that a nanovirus requires assembled viral particles for systemic infection
Source: PLoS Pathog. 2023 Jan 9;19(1):e1011086. doi: 10.1371/journal.ppat.1011086 (PMC9858847; doi:10.1371/journal.ppat.1011086)
Supplement: Attachment — Submitted filename: FBNSV_PPATHOGENS.3_Letter_with_responses_to_review_comments.pdf [file ppat.1011086.s008.pdf]

CBS — Centre de Biologie Structurale

INSERM U1054 – CNRS UMR 5048 – Université de Montpellier

Montpellier, December 21, 2022

Dear Editors

Please find attached our responses to the review comments on manuscript PPATHOGENS-D-22-01703 “*Structure-guided mutagenesis of the capsid protein indicates that a nanovirus requires assembled viral particles for systemic infection*”.

In order to respond to some of the comments, two additional experiments have been carried out. These are described in our responses and in the revised version of the manuscript (which includes now a modified version of S4 Fig).

We would like to thank all reviewers for their constructive comments and hope that our revised manuscript and the additional experiments meet all reviewers’ major criticisms.

With my best regards,

on behalf of all corresponding authors

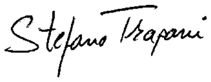

Stefano Trapani

associate professor

Université de Montpellier

Centre de Biologie Structurale – Inserm U1054, CNRS UMR 5048 , Univ Montpellier

29 rue de Navacelles - 34090 Montpellier

Tel: +33 (0)4 67 41 77 29 – Fax: +33 (0)4 67 41 79 13 – E-mail: stefano.trapani@cbs.cnrs.fr

Point-by-point responses to review comments

Please find here below our responses to all review comments.

We would like to thank all reviewers for their constructive comments and hope that our revised manuscript and the additional experiments described here below meet their major criticisms.

The reviews comments are in *italic gray font* (when they do not require a response) or *italic blue font* (when they require a response). Our responses are in upright black font. We have numbered our responses in order to easily refer to some of them. The indicated lines refer to the revised version of the manuscript.

In order to respond to some of the comments, two additional experiments have been carried out. These are described here below and in the revised version of the manuscript (which includes a modified version of S4 Fig).

> *Part I - Summary*
>
> *Please use this section to discuss strengths/weaknesses of study, novelty/significance, general execution and scholarship.*

> ***Reviewer #1:***

> *The manuscript entitled "Structure-guided mutagenesis of the capsid protein indicates that a nanovirus requires assembled viral particles for systemic infection" submitted by Trapani et al. is a very thorough study. The authors determine the structure of FBNSV and then use the structure to cleverly address a significant question regarding the infection and pathogenesis of a very multipartite plant virus. Nanoviruses are neither genetically nor biochemically tractable systems. The authors should be commended for their perseverance and creativity.*
>
> *Although this was the first time I saw the manuscript, I noted it was a resubmission. The document contained the authors' responses to four previous reviewers. To remain unbiased by the previous reviews, I first read the manuscript. Afterwards, I read the previous critiques and the authors' responses.*
>
> *I had no major concerns regarding the manuscript or the scientific rigor of the research, which I considered high. This is likely due to the comments of the previous reviewers. Below follows some minor points from my reading. After that, my comments regarding the previous reviews and the authors responses.*
>
> *Minor points:*
>
> *1. Line 82: Place (M-Rep) at the end of the sentence.*

Authors response N°1. The text has been modified accordingly (line 88).

> *2. Line 92-97: This run-on sentence would best be divided into two for clarity. I suggest, "A study on Faba Bean Necrotic Stunt Virus (FBNSV) [11] showed that a complete set of the eight genomic segments rarely occurs in individual cells of an infected plant. However, the function encoded by a given segment can complement the others at a distance, in cells where this segment is absent."*

Authors response N°2. The text has been modified accordingly (lines 98-102).

> *3. Line 98: replace either with "e. g."*

Authors response N°3. The text has been modified accordingly (line 104).

.

> *4. Line 104: FBNSV, and other multipartite viruses,*

Authors response N°4. The text has been modified accordingly (line 110).

5. Line 107-108: or alternatively as assembled viral particles.

Authors response N°5. The text has been modified accordingly (lines 113-114).

6. Line 122-125 I suggest the following rewording: The modified coat proteins could be expressed and accumulated into plant tissues; however, the long distance movement of the virus, and thus systemic infection of the host plant, was abolished in all cases.

Authors response N°6. The text has been modified accordingly (lines 128-130).

7. Line 143: I suggest: This heterogenous particle population, vis-a-vis DNA content, was then investigated by cryo-EM single particle analysis.

Authors response N°7. The text has been modified accordingly (lines 148-149).

8. Line 231-233: subordinate clauses with the word "which" are separated from the main part of the sentence with commas not parentheses. In fact, most of the parentheses in this paragraph should be commas.

Authors response N°8. The text has been modified accordingly (lines 234-239).

9. Legend to Figure S4: "inconsistently" not "unconstantly."

Authors response N°9. The text has been corrected (lines 950-951).

Comments regarding the previous reviews and the authors' responses

Firstly, I did not see the earlier version of this manuscript. Thus, my comments here reflect neither the quality of the review process nor the quality of the first submission, in which something may have been poorly explained. I read all of the comments from the last four reviews and the responses. When it was possible to respond positively, the authors made changes. In those instances when a positive response would require conducting experiments that are not feasible in this less than tractable system, the authors should not be penalized. As Otto Von Bismarck stated about politics, research is also the art of the possible. This must be considered when the research system is very intractable, as is the case with nanoviruses.

1. In the previous reviews, some of the reviewers requested experimental validation that the mutant coat proteins could not form capsids. Although the serine residues were very logical targets, this was a reasonable criticism. In this version of the manuscript, the authors provided that data. Thus, should no longer be a concern.

2. In the previous reviews, concern was expressed regarding the low efficiency of the wild-type infections. One reviewer expressed that it should be near 100%. Like the authors, I do not believe that positive controls need approach 100%. They need only set an upper limit and demonstrate how wild-type behaves in the same assay. The numbers given in Table 3 are quite compelling: 187/807 (~23%) of the plants used in the positive control developed symptoms, whereas 8/777 (~1%) of the mutant infections developed symptoms, but the wild-type coat protein gene, due to reversion, was detected in every one of those cases. Thus, it is <1/777 (0.1%). That is more than two orders of magnitude less than the positive control. Despite the low efficiency of the wild-type infections, the authors have rigorously demonstrated, by assaying almost 1600 plants, that the mutant coat proteins do not cause systemic infection. I agree with the authors responses on this point.

2. DNA binding studies: past reviewers stated that the manuscript should include DNA binding studies with wild-type and mutant coat proteins. In light of the in vitro work, which demonstrates that the mutant coat protein only forms pentamer, the results of such studies would be tangential, not altering the authors' conclusions regarding assembly competent capsids being needed for systemic infection. Moreover, without a clear understanding of nanovirus assembly and DNA packaging, the results of the experiment may be meaningless, or the experiment could not be conducted.

I searched PubMed for manuscripts on Nanovirus or Nanoviridae assembly and packaging, less than 10 manuscripts were identified, and the results of those manuscripts do not address the central question: do capsids assemble around the ssDNA genomes or are ssDNA genomes pumped into empty capsids? If the genome is pumped into a preformed capsid, the DNA interacting domains would be on the interior of the capsid and likely not available for nucleic acid interactions. If pentamers are the assembly subunit, as the data suggests, and is observed in other T=1 ssDNA viruses, one would need full length coat protein pentamers, which must include the positively charged N-terminus. However, the inclusion of this terminus leads to very little expression. Although this paper does contain some intriguing insights into assembly and packaging, the data can be interpreted to weakly support both established models. Moreover, nanovirus assembly is not the topic of this manuscript, and the in vitro assembly system may take years to develop. Thus, I agree with the authors' responses on this point.

3.Alternate hypotheses: several reviewers put forth alternative hypotheses. While these hypotheses could be occurring, they are far from the simplest hypothesis. For example, one such hypothesis states that the changes to the serine codons are acting on the DNA level, causing the segment to "behave badly." In this model, 1, 2, or 3 the altered nucleotides would affect a critical DNA structure or sequence substrate required to specifically move mutated DNA segment independently of an assembly competent coat protein. If this were the case, the sequence or a specific ssDNA structure was altered in making the mutations would be conserved in every genomic segment. Moreover, there is no evidence from the structure that the altered region of the coat protein is interacting with nucleic acid. Yes, this hypothesis is possible, but much more complex than the simplest hypothesis that the authors used to interpret their data. The authors do state in their manuscript, "We are perfectly aware that unpredicted effects of these mutations on the CP-DNA interactions cannot be totally excluded." I think that is sufficient.

4. Structure determination, ordered DNA and the lack of observable heterogeneity. I do agree with the reviewers when they state that the inclusion of more particles may be required to detect heterogeneity. However, considering the 3.2 Å resolution of the current model, these would be very subtle differences and would require a gargantuan effort, especially considering the less than tractable nature of the system. Moreover, greater resolution may not detect any differences. The organization of ssDNA within icosahedral capsids is extremely difficult to study. In most systems, a portion of the nucleic acid backbone is visible as ordered; however, distinct and recognizable nitrogenous bases are rarely, or have yet to be, observed. While not all systems are the same, my group has attempted several techniques including asymmetric reconstructions working with a virus for which purification yields particle concentrations of 1e13/ml. We have yet to see heterogeneity within the populations. We have attempted symmetric reconstructions with viable mutants that alter the path of the DNA during packaging that lead to dramatically difference solution properties. Although this did alter the pattern of the ordered DNA, the protein component of the structures did not differ from wild-type. The dynamics of virions in solution at 20°C are likely not reflected in liquid ethane, or in crystals. Importantly, the resolution of their structure was sufficient to identify mutations that would block particle assembly. I do not believe that analyzing more particles is likely to detect heterogeneity, and if it did, I do not see how it would change the interpretation of the biological assay: systemic infection requires an assembly competent coat protein. Eventually the authors may wish to look for different solution properties, if they can be detected, but developing the assays that could detect these differences will likely take years, especially since they do not have, or will ever have, a simple plaque assay. And lastly, it is not the topic of the manuscript.

Reviewer #2:

(These comments refer to Author summary)

What does 'several' mean?

Authors response N°10. We replaced "several" by "two or more" (line 50).

Please use 'spread' rather than 'propagate'

Authors response N°11. "propagate" replaced by "spread" (line 60).

Or concentrated in certain cells or tissues?

Authors response N°12. We are sorry, we do not understand what part of the Author summary is concerned by the referee's comment.

The last sentence doesn't make sense nor really reflect the results of the study-please delete or re-word

Authors response N°13. We have re-worded the sentence in the Author summary as follows (lines 66-73): "These results strongly suggest that the viral genome moves long distance as assembled viral particles. Such a situation, where all particles of the viral population are alike, does not support the existence of a large macromolecular complex which would form by specifically sorting and assembling particles each containing a distinct segment. Instead, our results, together with other evidence from totally unrelated approaches, further support the independent and random propagation of the distinct segments of the FBNSV genome."

Also, the last sentence of the Conclusion section is now reworded as two sentences (lines 520-526): "Such a situation, where all particles of the viral population are alike, does not support the existence of a large macromolecular complex which would form by specifically sorting and assembling particles each containing a distinct segment. Together with other evidence from totally unrelated approaches [11,45], this further supports the independent propagation of the distinct segments of the FBNSV genome; e.i. the random propagation described above."

We do hope that what we are trying to say is now easier to understand.

Reviewer #3:

The study by Trapani et al., "Structure-guided mutagenesis of the capsid protein suggests that a nanovirus requires formed viral particles for systemic infection," provides novel and intriguing information on FBNSV. The paper reports the structure of the virion using cryo-EM, experimentally confirms the importance of residues Ser 87 and Ser 88 in the formation of dimers, and hypothesises about how systemic spread works. Despite how challenging it is to deal with ssDNA viruses from nanoviruses, the authors have done an admirable job of uncovering their structure and propagation. The work is well done, and most of the conclusions are supported by the findings. All of the significant issues were brought up by the prior reviewers, and the authors have convincingly responded. The only point I want to raise is that the structural work on BBTV CP (ref. 27), which reported similar structural results, needs mention where the authors compare FBNSV with AYVV (pages 19, 20). Yes, a lot of unanswered questions remain, and I am sure the authors will take them up subsequently. However, the present work is no less significant, and it definitely deems publication in Plos Pathogen. I congratulate the authors on their tremendous efforts in comprehending nanovirus capsid assembly.

Authors response N°14. See author response N°22.

Reviewer #4:

This manuscript describes the determination of the cryoEM structure of the nanovirus, FBNSV, to near-atomic resolution. Though the authors anticipated that they might be able to observe differences in the structures of the particles encapsidating the different DNA components, this proved not to be the case. This is not particularly surprising given that all 8 cssDNA genomic segments are very similar in size (about 1kb) and must be able to interact productively with the coat protein. Nonetheless, the structure obtained is interesting and the methods used for its determination appear to be robust.

In addition to the straightforward cryoEM analysis, the authors also undertake mutagenesis experiments to identify amino acids important for the formation of capsids. In particular, they targeted S87 and S88 which are present at the two-fold axes. Introducing charged amino acids appears to negatively impact virus assembly and abolish or greatly reduce infectivity. In the case of reduced infectivity, revertants are found to accumulate. This is fine and is consistent with the amino acids being important for capsid formation. They then go on to postulate that this indicates that the virus moves in the form assembled particles. This is

> *where I think the manuscript goes a bit too far as I don't think their data actually addresses the proposed models of the mechanism of virus spread (the pluri-cellular way and the need for virus encapsidation). Given this I would rather the authors stick to the structural studies, coupled to mutagenesis of amino acids predicted to be essential for capsid formation, rather than stretch the story to include speculation about the entire replication cycle of the virus.*

Authors response N°15. With all respect due to the Reviewers' comments, and to how they greatly helped us improve the manuscript, we believe that there is a confusion on this specific point. Our earlier discovery that distinct FBNSV genome segments are located in distinct neighboring cells (the pluricellular way of life) implies that the product of the genes (mRNA and/or proteins) encoded on each segment have to traffic among these neighboring cells, to complement the various functions needed for the viral cycle at a supra cellular level (Sicard et al eLIFE 2019). Here we are specifically discussing on the movement of the segments themselves, not their product mRNAs or proteins (and we do not speculate about "the entire replication cycle of the virus").
It is very widely accepted that when a virus replicates its genome within host plant cells but cannot invade this host plant systemically, it is because it has a problem with cell-to-cell and/or long-distance movement. \
Here, we describe a FBNSV mutant that does replicate in the host plant cells (see Additional experiment 2 below) and produces a coat protein that can assemble into pentamers but cannot further organize these pentameric subunits to produce fully assembled viral particles. We further show that this mutant is unable to systemically invade the host plant and we propose that this is most likely because it cannot form particles. There is nothing heretic in this conclusion and we do not understand why Reviewer 4 requests the removal of our interpretation. With the additional experiments described below showing replication of all genomic segments, including the segment encoding the mutant coat protein, we would like to maintain this interpretation because i) we think it is a reasonable interpretation of the fact that the virus cannot systemically infect, and ii) it is simply consistent with common usage in the field.

> *Part II – Major Issues: Key Experiments Required for Acceptance*
>
> *Please use this section to detail the key new experiments or modifications of existing experiments that should be absolutely required to validate study conclusions.*
>
> *Generally, there should be no more than 3 such required experiments or major modifications for a "Major Revision" recommendation. If more than 3 experiments are necessary to validate the study conclusions, then you are encouraged to recommend "Reject".*

> ***Reviewer #1:***
>
> *None*

> ***Reviewer #2:***

> *1. My major concerns regarded the evidence of virion formation (or not) and this was a concern of the other reviewers. This seems even more important given the mutations are predicted to make virions unstable and this could be impacted in planta. This was partially addressed by the experiment showing E. coli-expressed mutant CP will not form full virions, whereas wild-type CP did. Nonetheless, the evidence still is somewhat circumstantial. Thus, it was disappointing that the ISEM method was so quickly dismissed as 'unimaginable' (Author response #16). Trying to scale it up would not be that hard. Moreover, the ISEM was effectively used for this purpose, i.e., correlating virion formation with systemic movement, for the monopartite geminivirus BCTV (Soto et al., 2005 Virology). It is fair to say that this approach has been tried, but was it shown there was differential replication in mesophyll vs phloem cells? What about aphid transmission-failure to be aphid-transmitted also would support lack of virion formation.*

Authors response N°16. Because the mutant virus does not infect the plant systemically, yes, scaling up is difficult. We can only use infiltrated leaves as biological sample where both wild type and mutated coat protein accumulate to low level, even more so with the mutated version (S4 Fig). What poses a major difficulty is the ratio between the amount of coat protein produced, so the possible amount of viral particles, over the total amount of plant material to be extracted. Scaling up would not change this ratio.

Additional experiment 1. We understand that Reviewer 2 wanted us to at least try the ISEM method before dismissing it as "unimaginable" and so we did for this second revision. To evaluate the feasibility, we first attempted a positive control. We collected the apex of plants infected systemically with the wild type FBNSV, because this is where the virus is mostly accumulated (Sicard et al 2013), ground the plants and try to capture virus particles on electron microscopy grids, coated or not with anti-coat protein IgGs. On both types of grids, among a variety of unidentified structures very variable in shape and size, we could observe some "spherules" of a size approximating 15-20nm in diameter. Unfortunately, because of the scarcity of such spherules, it would not be reliable (neither reasonable) to conclude that they were FBNSV particles. They could as well be undetermined cellular structures of different sorts. Consequently, we believe that ISEM approach cannot be applied in our case, because the mutated coat protein in infiltrated leaves is much less accumulated, and any reviewer could argue that not seeing virus particles in these conditions would never prove that they are indeed not assembled. Of note is also the fact that in the work cited by Reviewer 2 (Soto et al., 2005 Virology) the particles observed are characteristic of viruses of the family Geminiviridae and perhaps more easily identified because of their geminate structure. FBNSV particles are not geminate, they just appear as spherical structures in Transmission Electron Microscopy, that resemble many cellular structures, in particular proteasomes that are extremely numerous. To our opinion, the best alternative is the production of large quantities of coat protein via a bacterial expression system where the wild type coat protein forms pentamers that further assemble into virus particles, whereas the mutated coat protein assembly is arrested at the pentameric stage. We are sorry that Reviewer 2 considers this alternative as "circumstantial" but what we can now only say is that we have tried the direct ISEM approach, but we could not adapt it to the FBNSV, a very intractable system as acknowledged by Reviewer 1 and 3.

Finally, because the mutated version of FBNSV is not infecting host plants systemically, we do not see how aphid transmission experiment could be conducted. We understand that Reviewer 2 suggests that aphids may acquire the mutated version of the virus from infiltrated leaves (since it is not infecting systemic leaves). Then, would we observe a transmission failure, anyone could comment that it is not informative precisely because the mutated virus cannot systemically infect. This would be a sort of circular argument that we cannot solve.

> *2. I was puzzled that the authors chose not to use much of the literature of geminivirus long-distance movement. In multiple cases of monopartite geminiviruses evidence for the requirement of virions for systemic infection has been presented (MSV, BCTV), but for some bipartite species, the CP is dispensable. Thus, a non-virion small ssDNA virus can move in non-virion form and cause symptoms. The geminiviruses may also provide another model for long distance movement of nanoviruses: the 'waves of infection' of protophloem cells that ultimately give rise to mature sieve elements and their associated companion cells. This infection of the protophloem occurs through the Achilles heel of these progenitor cells developing into sieve element with large sized pores through which virions can pass and released ssDNA that enter the nucleus for replication of formation of more virions. The other way is mature companion cells are infected but various combinations of virions and this could happen via their unique deltoid plasmodesmata with a higher molecular size exclusion limit.*

Authors response N°17. We are sorry but we do not really understand this point. We know that some geminiviruses require the coat protein to move long distance while others do not. However, the main information of our manuscript is the atomic structure of the particle of a nanovirus, and we extensively discuss it in the context of the other known structures of viruses with a T=1 symmetry, geminiviruses and beyond. Additionally, we make use of the structural information to provide biological data suggesting that FBNSV does require full particle assembly to move/infect systemically. Going into further discussion on the mechanisms or pathways of movement of FBNSV in its host plant is out of the scope of the manuscript. We do not report experimental results allowing us to further speculate on this point. We would simply like to stick to the proposition that, whatever these mechanisms and pathways, the full assembly of virus particle appears mandatory for systemic movement.

> ***Reviewer #3:***
>
> *None*

Reviewer #4:

The major comment I have is that if the authors wish to examine whether the viral coat protein is necessary for the spread of viral genomic components, why not simply omit Segment S from the inoculum? If the coat protein essential for spread of the virus, inoculation with just the other 7 segments should not result in infection. Has this been attempted and, if so, what was the result? Making mutations with Segment S seems a much more convoluted way of investigating whether the coat protein is essential, particularly as point mutations are prone to reversion (which is observed). The other issue with the mutagenesis approach is that aberrant coat protein might have a dominant negative effect on the ability of the virus infection to spread. The formation of aberrant capsids could sequester the cssDNA and inhibit productive infection. A way of showing this not the case is to simply omit Segment S and compare the results with those found when wt or mutant S is included. I really do feel this is an essential experiment.

Authors response N°18. This requested additional experiment has already been done and published. Reviewer 4 is right on this point: omitting segment S at inoculation results in no infection. This has been previously published for two species of the genus nanovirus, FBNYV (Timchenko et al 2006, DOI 10.1099/vir.0.81753-0) and FBNSV (Grigoras et al 2018, https://doi.org/10.1016/j.virol.2018.07.001), the latter being the species used here.

Part III – Minor Issues: Editorial and Data Presentation Modifications

Please use this section for editorial suggestions as well as relatively minor modifications of existing data that would enhance clarity.

Reviewer #1:

(No Response)

Reviewer #2:

-I also agreed with reviewer #2 that DNA binding assays with wild-type and mutant proteins would be useful, as would showing replication of the mutant DNAs compared with wild-type.

Authors response N°19. About the DNA-Coat protein Binding assays, we are very convinced by the argument given by Reviewer 1 and quoted here: "*Without a clear understanding of nanovirus assembly and DNA packaging, the results of the experiment may be meaningless, or the experiment could not be conducted. I searched PubMed for manuscripts on Nanovirus or Nanoviridae assembly and packaging, less than 10 manuscripts were identified, and the results of those manuscripts do not address the central question: do capsids assemble around the ssDNA genomes or are ssDNA genomes pumped into empty capsids? If the genome is pumped into a preformed capsid, the DNA interacting domains would be on the interior of the capsid and likely not available for nucleic acid interactions. If pentamers are the assembly subunit, as the data suggests, and is observed in other T=1 ssDNA viruses, one would need full length coat protein pentamers, which must include the positively charged N-terminus. However, the inclusion of this terminus leads to very little expression.*" We cannot engage into elucidating this information gap in the context of this manuscript as this may take years of research, as it has clearly been the case in ssDNA microviruses for example, a literature we had to look into when planning to express the FBNSV coat protein in bacteria and to assemble VLPs.

Additional experiment 2: Concerning the need to demonstrate that the mutated FBNSV segment S replicates when agro-inoculated, we have performed additional experiments that are now reported/added in S4 Fig (lines 959-976)., in the Methods section (lines 648-664), and in corresponding text in the Results (lines 335-343) and Discussion (lines 458-461) sections. Leaves infiltrated with the 8 FBNSV cloned segments were

submitted to total DNA extraction and Rolling Circle Amplification (RCA), which specifically amplifies circular DNAs, so FBNSV replicated segments. As a negative control, the segment R encoding the replication protein M-Rep was omitted. While the RCA failed to amplify any FBNSV segments in the absence of segment R, we obtained positive amplification from the plants agro-inoculated with 8 segments, whether the segment S was mutated or not. Moreover, the accumulation of the mutated S segment within the RCA amplification products was estimated by qPCR. The presence of the mutation in segment S did not affect its accumulation compared to wild type.

> *-Regarding the infectivity data, I also share the concerns about low infectivity of the positive control but I agree with the authors that it is a very challenging experimental system and they address that with numbers of plants and replications. Regarding author response #7, it so happens agroinoculation of geminiviruses at least into common bean, cucurbit, tobacco and tomato routinely gives 100% infection and the key it delivery to the area of those protophloem cells just beneath the shoot apex, so it is possible.*
>
> *Of course nanoviruses have many more components and I agree that making a multimeric binary vector with all of them has been tried. Not sure about have 2-3/binary as it is possible to have at least a begomovirus and betasatellite on the same binary.*

Authors response N°20. We have had cloning problems as soon as we tried to assemble 2 or 3 distinct segments into the same plasmid. So eventually, 4 plasmids each containing two segments may be possible, or not, and whether this would enhance the infectivity is also unknown.

> *I agree with the authors on the silent mutation experiment, but I think the replication of the mutants, e.g., in a leaf disc assay should be demonstrated.*

Authors response N°21. We have performed such additional experiment for this second revision and it is described in the section “Additional experiment 2” above.

> ***Reviewer #3:***

> *The structural work on BBTV CP (ref. 27), which reported similar structural results, needs mention where the authors compare FBNSV with AYVV (pages 19, 20).*

Authors response N°22. The structural comparison of AYVV with the homology-based BBTV CP structure is now mentioned (lines 393-395).

> ***Reviewer #4:***

> *On a more minor issue, I felt that much of the material in the first paragraph of the "Discussion" would be better positioned in the "Introduction".*

Authors response N°23. Considering the logic of the narrative we have developed for presenting the results and their context in this manuscript, we do not agree with the reviewer on this point and we would like to maintain the organization of the discussion. Moving the first paragraph of the discussion into the introduction would require reorganizing the introduction and its logical flow as well.
